# Application of problem-based learning combined with a virtual simulation training platform in clinical biochemistry teaching during the COVID-19 pandemic

**DOI:** 10.3389/fmed.2022.985128

**Published:** 2022-10-26

**Authors:** Hongxiang Xie, Li Wang, Zhenzhen Pang, Sufeng Chen, Guoying Xu, Sumei Wang

**Affiliations:** ^1^Laboratory Medicine Center, Department of Clinical Laboratory, Zhejiang Provincial People's Hospital (Affiliated People's Hospital, Hangzhou Medical College), Hangzhou, China; ^2^Teaching-Research Office of General Practice, Department of Clinical Medicine, Hangzhou Medical College, Hangzhou, China; ^3^School of Medical Science and Laboratory Medicine, Institute of Medical Genetics and Reproductive Immunity, Jiangsu College of Nursing, Huaian, China

**Keywords:** COVID-19, PBL teaching, virtual simulation experiments, clinical biochemistry, DingTalk

## Abstract

**Background:**

The coronavirus disease 2019 (COVID-19) pandemic has had a great impact on the traditional teaching mode (Lecture-based Learning, LBL) and laboratory teaching. To address this challenge, the researchers conducted online Problem-based learning (PBL) teaching and virtual simulation laboratory teaching through DingTalk, and evaluated the effectiveness of this method in teaching clinical biochemistry.

**Methods:**

With the method of cluster sampling, the researchers randomly selected 60 students from two classes of the Class 2019 as the experimental group for this prospective experimental study. The theory class was taught online PBL through DingTalk, and experimental lectures were given by virtual simulation. After the experimental teaching, students were assessed for theory and operation. Self-administered questionnaires were administered through DingTalk. 65 students from our 2018 medical laboratory class were randomly selected as the control group, and offline LBL and traditional experimental teaching methods were used. Examination results were obtained through teaching portfolios.

**Results:**

The experimental group had significantly better examination scores in theoretical knowledge and experimental operational skills than the control group (87.45 ± 5.91 vs. 83.52 ± 9.94, *P* = 0.0095; 87.08 ± 12.42 vs. 80.18 ± 14.04, *P* = 0.0044). The results of the questionnaire survey revealed that the experimental group was more receptive to the DingTalk-PBL teaching method and virtual simulation laboratory teaching. Moreover, this hybrid teaching method was more effective in promoting basic knowledge understanding (95.0%, 57/60), facilitating the mastery of operational skills (93.3, 56/60), cultivating interest in learning (96.7%, 58/60), training clinical thinking (95.0%, 57/60), improving communication skills (95.0%, 57/60), and enhancing self-learning ability (91.7%, 55/60) and was more satisfying than traditional teaching method (all *P* < 0.05).

**Conclusion:**

The DingTalk-based PBL method combined with virtual simulation experiments was an effective and acceptable teaching strategy during the pandemic compared with the traditional teaching method.

## Introduction

With the emergence of coronavirus disease (COVID-19) in December 2019 and its rapid global spread, strict public health measures have been implemented worldwide (1). In response to the national requirement to “reduce nonessential gathering” for the prevention and control of the novel coronavirus pneumonia epidemic, medical schools in Mainland China have massively reduced offline face-to-face lectures and skills training courses (2). Moreover, many hospitals have implemented an outpatient appointment system, which has significantly reduced the number of inpatient admissions and prevented medical students from receiving internship and practicum training in hospitals. These factors have had significant effects on the traditional medical teaching method (3).

Traditional clinical biochemistry teaching is based on the traditional offline lecture method, that is, lecture-based learning (LBL); here, the teacher provides lectures, while the students listen. Clinical biochemistry is a discipline developed by blending chemistry, biochemistry, and clinical medicine. It is the main course of the laboratory medicine and a theoretical and practical discipline (4). Students are taught to understand and master biochemical metabolic concepts in normal states and biochemical changes in disease states, the basic principles of biochemical markers used for monitoring, and the selection, establishment, and evaluation of biochemical indicators for eventual clinical use. However, the LBL method has limitations in learning motivation, independent learning ability, and critical thinking exercises (5).

Problem-based learning (PBL) is a typical student-centered teaching method that places learning in complex, meaningful problem situations. Students work together in small groups to solve problems in the learning process and thus learn the scientific knowledge implied behind the problems to promote independent learning and the development of lifelong learning abilities (6). Studies have reported that PBL promotes knowledge assimilation, enhances critical thinking training, and helps improve the professional competence of the students (7). However, in the traditional PBL teaching method, students must get together in the classroom to discuss and share their opinions, making it difficult to implement and promote in clinical teaching, especially in the prevention and control phase of the pandemic.

To address this challenge and reduce the impact of COVID-19 on teaching, medical educators in various countries have actively explore new teaching methods. Social platforms such as Facebook and Twitter have been introduced to medical online education with great success (8, 9). Similarly, DingTalk is a free communication and collaboration multi-end platform with various features such as video conferencing, group check-in, posting assignments, and support for live recording and playback of courses. Given its powerful features, DingTalk has been widely used by major universities in China for online teaching during the pandemic (10, 11). DingTalk can eliminate the time and space constraints in medical education, and its effectiveness has been proven in flipped classrooms (12). However, only a few studies have evaluated the use of DingTalk combined with PBL in teaching clinical biochemistry.

Clinical biochemical belong to the field of medical technology, which involves various techniques and methods to analyze various chemical components in body fluid samples (4). Practical training operations and the clinical practice of clinical biochemistry are conducted throughout the teaching process, and the excellence or failure of the practical training environment is directly related to whether students can master the course well. As a result of the COVID-19, most schools have closed their laboratories or restricted their access, which has brought many difficulties to traditional experimental teaching. Virtual simulation (VS) experiment teaching can break the space and time constraints of traditional experiment, and provide a new idea to solve the above problems by constructing a highly simulated virtual experiment environment.

Virtual simulation technology is a three-dimensional virtual reconstruction of reality in a computer that gives users an immersive experience in multiple senses such as visual, tactile, and auditory, and enables human–computer interaction with good engagement and operability (13). VS has been widely used in medical teaching or training in surgery, dentistry, emergency, pediatric emergencies, basic medicine, medical radiography, puncture, and catheterization (14–17). Specifically, in undergraduate education, many studies have revealed that simulation-based learning (SBL) experiences help integrate theoretical knowledge with practice and acquire the skills needed for independent practice when learners respond in what they perceive as realistic settings (18–20).

In recent years, VS has received increasing attention as an emerging interactive teaching strategy in medical education (13). However, the effectiveness of VS in teaching laboratory medicine has barely been evaluated, and whether the online virtual experiment can achieve the expected teaching effect brings great challenges to practical teaching. In this study, the researchers aimed to evaluate the effectiveness of using a DingTalk-based PBL method in combination with VS experiments on online laboratory medicine teaching in the field of clinical biochemistry.

## Research methods

### Object of study

With the method of cluster sampling, 60 students from two classes of Class 2019 of medical laboratory majors were randomly selected as the experimental group for this prospective experimental study. Due to the continued outbreak of the COVID-19 in some regions, the return time of students of Class 2019 was postponed. The theoretical lectures were taught online by PBL through DingTalk, and the experimental operations were taught by VS. The 65 students from two classes of Class 2018 are also randomly selected by cluster sampling as the control group. The two classes have completed the traditional offline LBL and experimental studies in the school. The researchers retrospectively analyzed the theoretical and experimental results of these students with the teaching portfolios. All study participants had completed basic medical courses related to the testing profession, and all had a certain ability to use medical knowledge for comprehensive analysis. The two groups of students are taught by the same teachers, using the same syllabus and teaching materials In this study, no significant differences were found between the study participants regarding factors such as age, sex, and basic knowledge background.

### Teaching strategies

Experimental group: Theoretical lectures were taught using a DingTalk-based PBL teaching method. Students and teachers installed DingTalk on their cell phones or computers and created a DingTalk group. Teachers (*n* = 5) with ≥1 year of traditional PBL teaching experience were designated as teachers of the DingTalk-based PBL group. Moreover, 12 students and 1 teacher joined the same DingTalk group. The teacher selected typical teaching cases according to the teaching purpose, asked questions on key contents that were closely related to the clinic and some knowledge points that are difficult to understand and remember. For example, select representative and learnable diabetes cases, and organically combine them with the key and difficult parts of the syllabus, such as the detection of clinical biochemical indicators of diabetes and its clinical significance, and the biochemical differential diagnosis of type I and type II diabetes. The corresponding guiding questions will be designed according to the theoretical teaching content combined with the cases and posted them through the DingTalk group 1 week before the class. The students read the textbook or literature, make PowerPoint (PPT) presentations in groups, share relevant knowledge involved in the cases, and organize and summarize the questions raised by the teacher. During the class, the teacher and students interacted *via* video conference. Regarding class time, a student representative was selected as the facilitator to organize the presentations of the students in the group. After the PPT presentation of each group, other students were free to speak and ask questions at any time to discuss and complement each other. The teacher joined in the DingTalk-PBL discussion and occasionally gave guidance to students. The tutor's aim was to guide the group to think more broadly and deeply, and ensure that each group member was involved in the discussion. After the discussion, the teacher leads the students to conduct course feedback and summarize the corresponding knowledge points.

**Figure 1:**
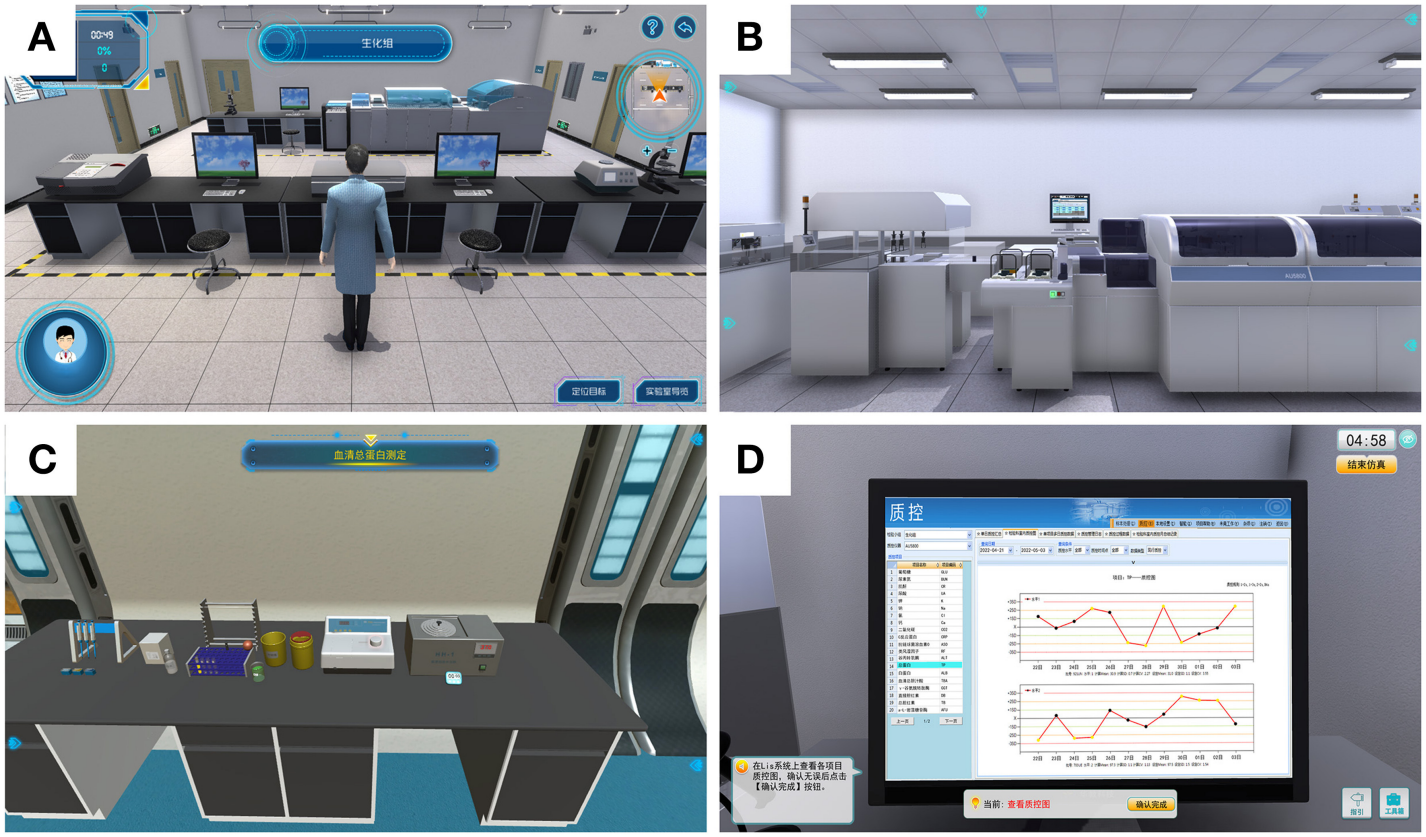
Virtual simulation laboratory component of the operating interface. **(A)** Virtual clinical biochemistry laboratory. **(B)** Automatic biochemical analysis of the virtual simulation system. **(C)** Total serum protein and serum glucose determination. **(D)** Clinical biochemistry assay and quality-control virtual simulation platform.

The VS laboratory system is used for online teaching in laboratory courses. The system has a student operating station and a teacher checking and guiding station. After entering the platform, the teacher performs demonstrations to the students by live streaming, performing step-by-step interpretation, and simulation operations. Students can practice by entering the platform through their student accounts. In the demonstration mode, students can learn to browse relevant experimental processes, and in the operation mode, they can individually practice specific operations and make a real-time assessment of their operating results. Through the virtual experimental project, students can master the experiments and essential actions of the classic manual test project. Through the virtual test instrument, they can master the internal structure, operation principle, quality control, calibration, sample detection, reagent replacement, maintenance, and alarm handling of the automatic biochemical analyzer. Part of the operation interface of the virtual simulation laboratory is shown in Figure 1. The teacher can observe the accuracy and standardization of the students' operation process through DingTalk screen sharing, provide guidance, and access the system through the teacher's account to view the students' operating time, proficiency, and results. Moreover, the platform will also automatically count major errors in the procedure, and the teacher will explain the common errors after the class.

Control group: Theoretical lectures were taught by the traditional LBL method, with classroom lectures as the main focus, and the teacher taught the basic knowledge and basic theory of the clinical biochemistry course according to the syllabus. In the experimental class, the teacher taught the purposes, principles, methods, operation points, and precautions of the experiment. As the students practiced in groups, the teacher went around and guided them. After the experiment, students wrote the laboratory report as required, and the teacher corrected it.

### Student assessment

Student assessment was divided into two parts: The first part referred to evaluation after the teaching, including theoretical and laboratory tests to evaluate the teaching effect. The theoretical test was assessed in the form of a closed-book examination, including basic theoretical knowledge assessment and clinical case analysis. For the experimental test, students randomly selected experimental items and completed the test parameter setting and test procedure operation. The examination results of the control group were taken from the teaching portfolios. for data analysis. Second, questionnaires were used to collect perceptions of the students. Three questionnaires were administered in this study. Questionnaires A and B investigated students' acceptance of the DingTalk-based PBL teaching combined with VS experiment teaching. Questionnaire C investigated the overall teaching satisfaction of the students in experimental group 1 and control group. The questionnaires were designed and modified from previous studies and had features specific to this study, with their validity verified in previous study and determined through several discussions and revisions by the three subject experts in departments of medical education in our study (21, 22). The reliability of the questionnaires was evaluated using internal consistency; the Cronbach alpha coefficient were 0.911, 0.855, 0.928, respectively. Informed consent was obtained from all participants prior to data collection.

### Statistical data

Data analysis was performed using SPSS 26.0 (IBM Corp., Armonk, NY, USA). Continuous variables were expressed as means and standard deviations, and frequencies and percentages were used for categorical variables. Independent *t*-test was performed for continuous variables such as age and test scores (normal distribution). Chi-square tests were used to analyze categorical variables such as sex (male/female) and satisfaction (satisfied/unsatisfied). Differences were considered significant when *p* < 0.05.

## Results

### Comparison of course grades

The study enrolled a total of 125 students, which included 60 students in the experimental group and 65 in the control group. No significant differences were found between the two groups in terms of age and sex at baseline (Table 1). After the semester, the experimental group had significantly higher scores on the theoretical examination (87.45 ± 5.91 vs. 83.52 ± 9.94) and practical skills test (87.08 ± 12.42 vs. 80.18 ± 14.04) than the control group (Table 2).

**Table 1:** Basic information of the two student groups.

	**Experimental group** ** (*n =* 60)**	**Control group** ** (*n =* 65)**	***t*/*z* value**	***P-value***
Age	20.35 ± 1.102	20.58 ± 1.158	1.158	0.2490
Sex			0.7215	0.4706
Male [n (%)]	25 (41.67%)	23 (35.38%)		
Female [n (%)]	35 (58.33%)	42 (64.62%)		

**Table 2:** Comparison of theoretical and practical test scores between the two student groups.

	**Experimental group (*n =* 60)**	**Control group** ** (*n =* 65)**	***t-*value**	***P-value***
Theoretical test	87.45 ± 5.91	83.52 ± 9.94	2.622	0.0095
Practical operation	87.08 ± 12.42	80.18 ± 14.04	2.900	0.0044

### Survey on the acceptance of the DingTalk-based PBL teaching method

At the end of the semester, Questionnaire A was sent to students in the experimental group *via* DingTalk to assess the acceptance of the DingTalk-based PBL teaching method. In this study, 60 questionnaires were distributed, and 60 were returned, with a valid return rate of 100% (Table 3). The results of the questionnaire survey revealed that the DingTalk-based PBL teaching method achieved positive results. Most students liked DingTalk for online PBL teaching (46/60, 76.7%), found it valuable and interesting in terms of the quality of clinical cases (56/60, 93.3%), were satisfied with the teaching resources provided by the platform (56/60, 93.3%). Moreover, the majority of the students rated other aspects of the method highly, including completing assigned tasks on time (55/60, 91.7%), applying prior knowledge to solve problems (55/60, 91.7%), and critically evaluating acquired data (53/60, 88.3%). The majority of the students (56/60, 93.3%) believed that the teacher demonstrated continuous enthusiasm in online teaching and that the teacher provided timely feedback to improve their learning (55/60, 91.7%).

**Table 3:** Questionnaire survey results of the DingTalk-based PBL teaching method.

**Questions**	**Yes** ***n* (%)**	**No** ***n* (%)**
1. Do you like to discuss the PBL case?	46 (76.7)	14 (23.3)
2. Do you like online PBL teaching through DingTalk?	44 (73.3)	16 (26.7)
3. Can you share ideas as effectively as possible in a group discussion?	50 (83.3)	10 (16.7)
4. Can you complete your tasks on time?	55 (91.7)	5 (8.3)
5. Can you apply your learning to solve the problem?	55 (91.7)	5 (8.3)
6. Did you notice the intrinsic connection of the information?	53 (88.3)	7 (11.7)
7. Can you critically evaluate the knowledge you have gained?	53 (88.3)	7 (11.7)
8. Are your teachers enthusiastic about teaching PBL?	56 (93.3)	4 (6.7)
9. Do your group members and teachers provide timely feedback to enhance your learning?	55 (91.7)	5 (8.3)
10. Do you find the mini-clinical cases presented in PBL valuable and interesting?	56 (93.3)	4 (6.7)
11. Are you satisfied with the teaching resources provided by the PBL teaching method?	56 (93.3)	4 (6.7)
12. Do you think DingTalk is an effective tool for teaching PBL in clinical biochemistry?	53 (88.3)	7 (11.7)
13. Do you agree that the online DingTalk-based PBL teaching method can improve clinical competence?	56 (93.3)	4 (6.7)

n, the numbers of those students who agreed to the statements provided in the left.

### Survey on the acceptance of VS experiment teaching through DingTalk

Table 4 displays students' feedback on the virtual laboratory. The vast majority of students found the method useful (57/60, 95.0%) and enjoyable (55/60, 91.7%). While performing the exercises, almost all students felt comfortable communicating with the teacher by voice or video through DingTalk (54/60, 90.0%). Many found the virtual format to be more authentic than the traditional format (54/60, 90.0%). Compared with laboratory-based instruction, 86.7% of the students felt more “engaged” during the VS. Moreover, 93.3% of the students reported that the virtual laboratory improved their understanding of the concept. 93.3% of the students reported that the virtual exams were fair and objective. The majority (85.0%) of the students indicated that more VS should be used in future laboratory courses, which would help them prepare for future internships. As regards technical issues, 91.7% of the students reported that the virtual laboratory was easy to use. Students also found the interface of the virtual laboratory user-friendly and easy to log in, as they can navigate through the laboratory using the physician avatar and locate examination rooms according to the instructions. Moreover, 93.3% of the students reported that the virtual laboratory (images and sound) had excellent quality. Only four students (6.7%) experienced mild discomforts, such as dizziness, headache, or nausea, while viewing and operating the virtual laboratory.

**Table 4:** Findings of DingTalk-based virtual simulation teaching.

**Question**	**Agree** ***n* (%)**	**Disagree** ***n* (%)**
1. The virtual simulation laboratory system is useful.	57 (95.0)	3 (5.3)
2. The DingTalk-based virtual simulation experiment teaching was pleasant.	55 (91.7)	5 (8.3)
3. During operation, I can communicate comfortably through DingTalk.	54 (90.0)	6 (10.0)
4. Virtual laboratory classes offer a more realistic experience than traditional laboratory ones.	54 (90.0)	6 (10.0)
5. Compared with traditional experimental classes, I was more actively involved in the virtual laboratory classes.	52 (86.7)	8 (13.3)
6. The virtual laboratory contributes to my learning experience of clinical biochemistry and increases my knowledge.	56 (93.3)	4 (6.7)
7. The virtual laboratory simulation examination is more fair and objective than the traditional experimental examination.	56 (93.3)	4 (6.7)
8. I prefer to use virtual laboratories to complete more testing projects than traditional laboratories.	51 (85.0)	9 (15.0)
9. I want to use this teaching method to prepare for my future internship.	51 (85.0)	9 (15.0)
10. The virtual simulation laboratory system is easy to use.	55 (91.7)	5 (8.3)
11. Logging into the virtual laboratory is easy following the instructions provided.	56 (93.3)	4 (6.7)
12. Navigating through the virtual laboratory is easy using my avatar.	55 (91.7)	5 (8.3)
13. The video and audio quality was excellent.	56 (93.3)	4 (6.7)
14. I can comfortably look around the laboratory and perform experimental operations without any discomfort.	56 (93.3)	4 (6.7)

n, the numbers of those students who agreed to the statements provided in the left.

### Student assessment

After the study, Questionnaire 3 was distributed to students in the experimental and control groups, and 125 questionnaires were returned, with a 100% return rate. The survey results revealed that the students in the experimental group were interested in “basic knowledge understanding,” “operational skills mastery,” “learning interest cultivation,” and “clinical thinking.” Moreover, the survey results revealed that the experimental group was more satisfied than the control group in terms of “basic knowledge understanding,” “operational skills mastery,” “learning interest development,” “clinical thinking,” “communication skills improvement,” and “self-learning ability enhancement,” and the difference was significant (*P* < 0.05) (Table 5).

**Table 5:** Comparison of students' satisfaction with the teaching effect between the two groups.

	**Experimental** ** group % (*n*/60)**	**Control** ** group % (*n*/65)**	***P-value***
1. It helps understand rudimentary knowledge	95.0 (57/60)	81.5 (53/65)	0.0269
2. It helps master operating skills	93.3 (56/60)	75.4 (49/65)	0.0071
3. It helps deepen interest in learning	96.7 (58/60)	69.2 (45/65)	< 0.001
4. It helps the training of clinical thinking	95.0 (57/60)	63.1 (41/65)	< 0.001
5. It helps improve communication skills	95.0 (57/60)	56.9 (37/65)	< 0.001
6. It helps enhance self-learning abilities	91.7 (55/60)	43.1 (28/65)	< 0.001

Informal interviews were also conducted with the participants to further understand the limitations of this hybrid teaching method in teaching clinical biochemistry, and a total of 11 students completed the interviews. The main reasons for students' dislike of this hybrid teaching method were as follows: (1) Online PBL requires much time and energy, requiring the use of personal breaks to review a large amount of information in addition to regular classes and focused discussions. (2) Unlike LBL, the information in online PBL lacks focus and organization; thus, greater effort is needed to filter and extract valid information. (3) Compared with traditional laboratory teaching, simulated clinical laboratory scenarios lack realism, and discomfort such as loneliness and dizziness can occur with prolonged online operation. (4) It is affected by a poor network connection.

## Discussion

The rapid development of computers and the Internet has driven the transformation in medical education. At present, more and more medical schools worldwide choose to use online-teaching websites to set up courses as a tool to assist students' learning. In 2018, the Chinese Ministry of Education launched the “Education Informatization 2.0 Action Plan” to facilitate the transformation of education informatization toward the Internet (23). The COVID-19 pandemic and quarantine measures taken by the Chinese government have accelerated the transformation of online education, and major medical schools in China are actively looking for innovative educational methods to replace traditional classroom education (24).

PBL has been widely used as a modern teaching method in undergraduate medical education (25). Rote memorization of theoretical knowledge is no longer the learning goal of students, but the practical application of mastering theoretical knowledge. However, in China, PBL has not been fully implemented and promoted because of limitations in manpower, time, and location. Educators have tried to optimize traditional PBL by improving teaching methods such as using flipped classrooms and interprofessional education combined with PBL (26, 27). Despite the great progress in addressing the shortage of teachers, effective solutions to break the geographical and temporal constraints of effective courses are still lacking.

In recent years, it is believed that the DingTalk may break through this limitation and benefit online medical education during the COVID-19 pandemic (28). DingTalk is one of the most common online communication platforms in China, and currently, it is used by 500 million people and 19 million organizations. DingTalk was believed to help improve the efficiency of medical education (29). In this study, used an online DingTalk-based PBL method to compensate for the time and space limitations in traditional PBL while maintaining its advantages.

Digital PBL is as effective as and more effective than traditional PBL in improving knowledge (30). The traditional teaching of clinical biochemistry is focused on theoretical teaching and lacks the integration of basic knowledge. When the researchers teach PBL online, the researchers ask students to explain the mechanism from the beginning when answering the guiding questions and encourage them to integrate their previous knowledge of analytical chemistry, pathophysiology, and biochemistry to help them understand the “why.” This has largely made clinical biochemistry more of a medical discipline than a technical one. Second, most of the online PBL cases were selected directly from clinical work in the laboratory. The authenticity of the learning materials stimulates students' interest in exploring and solving problems, which truly integrates theory and practice and helps maintain students' motivation and drive.

Compared with traditional PBL teaching method, our DingTalk-based PBL teaching method has several distinct advantages. First, online PBL is more efficient and diverse in terms of case preparation. Clinical cases are uploaded to the DingTalk group before class, and students can prepare for the course in advance. Group members can share valuable information in the group chat, including texts, videos, audios, and images. This real-time sharing of information can effectively improve the efficiency and quality of online PBL discussions. Second, the DingTalk group establishes a regular connection between teachers and students. The screen sharing in the DingTalk group promotes interaction among students and teachers and encourages timely feedback from students about the course. Third, compared with traditional PBL, DingTalk-based PBL can encourage more teamwork, as students can easily communicate with the help of the program. Fourth, the statistics feature of DingTalk allows teachers to have a clear understanding of students' learning, such as the number of visits, viewing hours, and openings of a certain video. To a certain extent, attendance, fixed assignment submission time, time limit test, etc., can play a positive role in students' learning. Finally, the platform provides a playback function, which facilitates students to review after class.

However, the effectiveness of PBL in improving students' performance is complex, which depends on many factors, such as students' preparation, course content, implementation and evaluation of PBL (31, 32). Students' potential resistance to PBL should be considered prior to its implementation. PBL requires students to change their learning mentality and behavior, which is very challenging for many students who are accustomed to the traditional teaching mode. Teachers should always realize the frustration associated with PBL (31, 32). Although the same syllabus and materials are used in the teaching, the researchers lack an assessment of whether there are differences between different teachers. Different from the traditional teaching PBL adopts the “small class” teaching mode, and a teacher faces about 10 students, thus giving more attention to students than the traditional LBL teaching in which a teacher faces more than 30 students. It is worth discussing whether this will cause the difference in teaching effect. In addition, due to the COVID-19, the traditional offline courses could not be carried out simultaneously during the online teaching, so the students of the same major admitted in the previous year were selected in the control group this time, and there might be differences in academic levels between the two groups of students.

The teaching content in clinical biochemistry includes both theoretical teaching and experimental teaching, and practice and operation play crucial roles in teaching (33). Therefore, online PBL alone cannot meet students' demand for practical skills. Traditional experimental teaching has disadvantages, such as very limited experimental instruments and sites, too traditional and single content, and a big difference with clinical testing technology. VS has the advantages of having flexible teaching sites, rich teaching contents, video and standardization of instruments and operation methods, learners are not restricted by time and space, etc. The learning effect achieved by VS experiments can be equal to or even better than the effect achieved in the real environment (34). With modern developments in computer and Internet creating opportunities for remote simulated virtual experiences, SBL has shifted online and is accessible through web browsers (35).

As a whole, our clinical biochemical test VS laboratory adopts Browser-Server (B/S) architecture, using virtual reality (VR) technology and virtual interactive animation for software development, with a web browser as the system running the platform without the need to install other software, such as PC, cell phone, and iPad, and can be accessed to avoid limitation to time, space, or equipment. The teaching platform is centered on the requirements of internship, employment, and job competence and mainly contains modules, such as virtual biochemistry, automated instrument operating system, virtual experiment, and clinical thinking training system. The automated operating system mainly introduces various biochemical instruments commonly used in clinical biochemistry (such as automatic biochemistry analyzer, electrolyte analyzer, chemiluminescence analyzer, and blood gas analyzer), including the structure, principles, precautions, and results of the analysis of each instrument, which facilitates students' full understanding and mastery of the experimental content. The virtual experiment function module consists of two modules: classical manual test items and design experiments. The former exhibited the whole experimental process from the perspectives of principles, methods, operations, result analysis, etc. The latter makes full use of the intelligent, simulation, image, and fun features of the virtual experiment system; thus, students can design and conduct corresponding experiments, have fully mobilize interest in learning, and cultivate their creativity through operation interaction and prompting. There are clinical thinking training modules such as disease-centered project inspection, analysis and solutions of the process of internal quality control going wrong, they can help further improve students' experimental diagnostic thinking ability, test result analysis ability, and clinical communication ability. Moreover, online virtual laboratory teaching with DingTalk allows teachers and students in scattered locations to “share” the same virtual space and participate in and collaborate to complete the design or training of certain projects. Information is transmitted in two or even multiple ways, which is appropriate for teaching interaction and cultivation of students' sense of collaboration.

Since virtual experiment teaching breaks geographical and temporal restrictions, students can also arrange learning time outside the class and choose the required learning content independently, without being restricted by the opening hours of a real laboratory (13). In this study, students in the experimental group who received VS teaching performed significantly better than the control group. This is because the difficulty of experimental skills operation lies in the memorization of standard steps and procedures, and the VS project can reproduce the standard steps in experimental skills operation completely. Further, the researchers found a significant correlation between students' online operation hours and their test scores.

The hybrid DingTalk-based PBL teaching method combined with VS experiments faces certain challenges. First, large-scale online teaching in universities often suffers from network congestion or crashes, which affect teaching quality (28). Remote or rural areas often do not have stable network connections, and students in these areas may need hardware and information technology (IT) support to participate in online classes. Essentially, the widespread use of 5G telecommunications technology can solve these problems because it can provide high-quality and high-capacity transmission for live streaming and interaction (36, 37). Second, the method imposes certain requirements on the information technology literacy of both students and teachers. Students should pay attention to IT courses. Teachers should not only change their teaching philosophy but also strengthen their Internet knowledge base. Moreover, online PBL and VS teaching put higher demands on the self-discipline and time management skills of the students. High-quality teaching emphasizes the balance of freedom and discipline, and real-time supervision of the teaching process remains one of the key challenges of online teaching (38). In addition, online VS experiment terminals are computers and cell phones operated by a mouse and have touchscreen features, which are still different from real skill operations. Since being in a virtual environment for a long time may cause loneliness and depression, teachers should use resources wisely and invest more humanistic care for students during the teaching process (38).

In conclusion, this study presents that DingTalk, which is one of the most widely used instant messaging applications in China, provides a good interactive platform for teaching clinical biochemistry. The hybrid teaching method of DingTalk-based PBL combined with VS experiments is an effective way to teach laboratory medicine during the pandemic and may provide new teaching strategies for other clinical disciplines.

## Data availability statement

The datasets analyzed in this study are available from the corresponding authors on a reasonable request.

## Ethics statement

The studies involving human participants were reviewed and approved by the Ethics Committee of Jiangsu College of Nursing. Written informed consent from the participants was not required to participate in this study in accordance with the national legislation and the institutional requirements.

## Author contributions

HX: funding acquisition and writing of the original draft. ZP, LW, and SC analyzed the data and revising the manuscript. SW reviewed and edited the paper. GX: funding acquisition, collected and analyzed the data, and prepared the tables and figures. All authors read and approved the final manuscript.

## Funding

This work was supported by Medical and Health Science and Technology Program of Zhejiang Province (2021KY017 and 2022KY027), Educational Committee Program of Zhejiang Province (Y202146133), Traditional Chinese Medicine Science and Technology Program of Zhejiang Province (2023ZL009) to HX, Key Project of Jiangsu Vocational and Technical Education Society (XHZDB2021004), and Jiangsu Province Higher Education Teaching Reform Research Project (2019JSJG475) to GX.

## Conflict of interest

The authors declare that the research was conducted in the absence of any commercial or financial relationships that could be construed as a potential conflict of interest.

## Publisher's note

All claims expressed in this article are solely those of the authors and do not necessarily represent those of their affiliated organizations, or those of the publisher, the editors and the reviewers. Any product that may be evaluated in this article, or claim that may be made by its manufacturer, is not guaranteed or endorsed by the publisher.
